# Surgical-experimental principles of anterior cruciate ligament (ACL) reconstruction with open growth plates

**DOI:** 10.1186/s40634-015-0027-z

**Published:** 2015-05-10

**Authors:** Romain Seil, Frederick K Weitz, Dietrich Pape

**Affiliations:** Department of Orthopaedic Surgery, Centre Hospitalier Luxembourg, Clinique d’Eich. 78, rue d’Eich, L-1460 Luxembourg, Luxembourg; Sports Medicine Research Laboratory, Luxembourg Institute of Health, 78 rue d’Eich, L-1460 Luxembourg, Luxembourg; Department of Pediatric Surgery, University of Tampere, Teiskontie 35, 33521 Tampere, Finland

## Abstract

**Objective:**

To review surgical and animal experimental studies performed with open growth plates in relation with pediatric anterior cruciate ligament (ACL) reconstruction.

**Backround:**

When it comes to the treatment of ACL injured children, there is a lack of current international guidelines, leaving the treating physicians with a therapeutic dilemma. A variety of surgical and animal experimental studies have been undertaken over the last decades in relation with open growth plates and ACL-reconstruction.

**Method:**

Based on our own previous animal experimental data, we highlighted 15 specific points concerning pediatric ACL-reconstruction and reviewed additional literature concerning these individual items.

**Results:**

Pediatric ACL-reconstruction could be proven to be safe in animal models. Growth abnormalities, risk factors and factors, which were specifically related to biological healing processes in children, were identified. From them surgical principles for safe pediatric ACL replacements can be deducted. Applying these principles through a correct technical execution of surgery may prevent clinically significant growth changes.

**Conclusion:**

Over the last 2 decades it has been shown that a technically correct pediatric ACL reconstruction has little risk in creating clinically significant growth abnormalities. Animal experiments support this hypothesis despite the fact that the gained knowledge cannot be fully generalized to humans. More long time follow-up is needed to fully understand the complete risk factors related to ACL surgery with open growth plates.

**Electronic supplementary material:**

The online version of this article (doi:10.1186/s40634-015-0027-z) contains supplementary material, which is available to authorized users.

## Introduction

Anterior cruciate ligament (ACL) injuries are among the most frequent severe knee injuries affecting physically active children and adolescents. They have both short and long-term consequences, affecting future knee structure and function with subsequent meniscus and cartilage injuries, early development of osteoarthritis and a serious economic burden to civil society due to potentially shortened professional careers and later surgeries. McConkey et al. (2011) reported that 0.5-3% of all ACL injuries occur in children and adolescents and the incidence is increasing due to increased sports participation. Shea et al. (2004) reviewed the insurance claims in football over a 5 years period in athletes aged from 5 to 18. Of 8215 total claims, knee injuries accounted for 22% and from the knee claims ACL injuries accounted for 31%. At the end of the growth period, the incidence of ACL injuries increases dramatically, reaching a peak in the group of 15–20 years old female athletes who participate in sports that require sudden deceleration, landing and pivoting movements (Engelman et al. 2014; Arendt and Dick 1995; Yu and Garrett 2007). Altogether, ACL injuries in children, adolescents and young adults are increasing (Aichroth 2002; Mohtadi and Grant 2006). The main reasons for this are participation in organized youth sports, increased female participation in high risk sports, potentially decreased motoric skills in this population and the physicians’ improved diagnostic skills.

**Figure 1:**
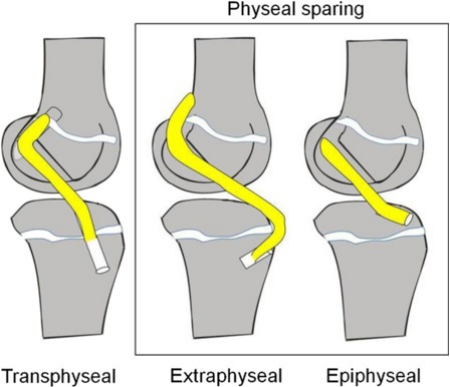
Representation of different pediatric ACL reconstruction techniques in lateral knee views. Surgeons differentiate between transphyseal and physeal sparing techniques. The former implicate drilling of a bone tunnel through the femoral and tibial growth plate whereas the latter do not cause any direct iatrogenic physeal injuries, but bear the risk of indirect damage to the growth plate. The ACL grafts are placed either within the epiphysis or turn around the physis. Many surgeons use different techniques on the femoral and the tibial side.

When it comes to the treatment of ACL injured children, there is a lack of current international guidelines, leaving the treating physicians with a therapeutic dilemma. On the one hand, nonoperative treatment has shown to be successful in some patients (Woods and O'Connor 2004; Moksnes et al. 2013), but the reasons for this are poorly understood (Funahashi et al. 2014). Furthermore, a strong association between the delay of surgery and the occurrence of meniscus and cartilage lesions strongly suggests that a nonoperative treatment may be detrimental to the intra-articular soft tissues. On the other hand, surgery is difficult and highly specialized due to the specific anatomy of children’s knees and its serious complication potential. Therefore, anterior cruciate ligament replacement surgery in children is controversial and many operative techniques have been described (Figure 1). All of them bear a specific risk for growth disturbances, either through indirect growth changes in extraepiphyseal surgical procedures or through growth plate injuries in epiphyseal or transphyseal techniques (Mohtadi and Grant 2006; McConkey et al. 2011; Kocher et al. 2007; Frosch et al. 2010; Lo et al. 1997).

Every surgical procedure or injury near to growth plate may lead to growth abnormalities. Experimental studies on the growth plate cartilage helps understanding the risk of such growth changes (Ollier 1867; Bidder 1873; Haas 1945a,b; Ford and Key 1956). The purpose of the present review article is (1) to illustrate the surgical and experimental knowledge on growth plate injuries related to pediatric ACL reconstruction and (2) show the relation between routinely used tibial and femoral tunnels of an intra-articular, transphyseal ACL replacement technique, the tibial and femoral growth plate and relevant soft-tissue structures around the knee.

### Anatomy and function of the growth plate

**Figure 2:**
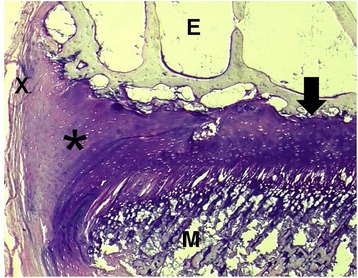
Distal femoral growth plate of a 10 months old sheep (x = perichondral fibrous ring of LaCroix; * = ossification groove of Ranvier; E = epiphysis; M = metaphysis; arrow = center of the growth plate with columnal chondrocyte structure) (Giemsa staining; magnification x 25).

The growth plate is located between the epiphysis and the metaphysis of long bones. It regulates endochondral growth and has in its center a complex anatomy with the following cellular layers (from the epiphyses to the metaphysis): the reserve zone, the proliferative zone, the layer with prehypertrophic chondrocytes and the hypertrophic zone which is subdivided in cellular layers of maturation, degeneration and calcification (Jouve et al. 1998; Mackie et al. 2011) (Figure 2). The review does not intend to give a detailed overview of the cellular and molecular levels of the growth plate cartilage, recently described in details by Mackie et al. (2011) and Michigami (2013). Vascularisation is separated between the epi- and the metaphysis and as a consequence the growth plate represents a frontier between these 2 structures playing an important role in the physeal pathology of certain tumors and infections. At its periphery we find the presence of the perichondral structures. They are composed by the perichondral ring of Lacroix (Lacroix 1951) providing mechanical support and the ossification groove of Ranvier (Ranvier 1889) which provides cells for growth in width. In a newer study, Karlsson et al. (2009) exposed 3 month old New Zealand white rabbits to bromodeoxyuridine (BrdU). They could find BrdU positive cells i.e. proliferative cells in the perichondral zone of Ranvier and in all zones of cartilage. After wash out, a small amount of progenitor cells were present in the cartilage and in the perichondral groove of Ranvier, showing that the latter is also a stem cell niche. They have an important physiologic role thus constituting the crossroads between longitudinal growth and growth in width of the bone. Furthermore, their presence plays an important role in the stabilization of this transitional zone between the epi- and the metaphysis. The perichondral structures do also play an important role from a pathologic point of view through the specific pediatric fractures classified as #6 in the Ogden classification system (Ogden 1981).

### Experimental principles on surgery with open growth plates

The question of remaining endochondral growth after growth plate injuries has been of great interest for orthopaedic surgeons for more than 150 years. Already in 1867 Ollier performed surgical experiments on cats in which he sectioned the growth plate longitudinally. He noticed that the superficial sections did not influence longitudinal growth whereas deep cuts did induce growth abnormalities. Haas noted in 1945 that a cerclage wire which was slung around the growth plate resulted in a decrease of longitudinal growth. In case of a breakage of the wire, growth continued normally. Based on the first clinical experiences with epiphysiodesis (Phemister 1933; Blount and Clarke 1949) in the first half of the 20^th^ century, many experimental studies were published with the goal to develop treatments regulating longitudinal growth like temporary epiphysiodesis, epiphysiolysis capitis femoris and their respective fixation principles (Haas 1945a,b, 1950; Campbell et al. 1959; Friedenberg 1957; Ford and Key 1956; Key and Ford 1958; Siffert 1956; Barash and Siffert 1966; Nordentoft 1969). With the development of ACL reconstruction techniques and the identification of the problem of ACL injuries in children, several specific surgical-experimental studies analysing paediatric ACL replacements were published during the last 3 decades. They were conducted in rabbits, pigs, sheep and dogs with open growth plates (Stadelmaier et al. 1995; Guzzanti et al. 1994; Janarv et al. 1998; Ono et al. 1998; Edwards et al. 2001; Seil et al. 2008; Meller et al. 2008a,b, 2009a,b, Chudik et al. 2007; Murray et al. 2010). They allowed recognizing the risks related to specific surgical techniques and especially the fact, that a technically correct anterior cruciate ligament surgery in a pediatric patient bears little risk of a clinically relevant secondary growth change. From these experimental studies on the growth plate as well as clinical experiences from the past, a certain number of surgical principles can be applied either directly or indirectly to ACL reconstruction with open growth plates. The surgical principles, which will be discussed in this review paper, are represented in Table 1.

**Table 1:** **Surgical-experimental principles of pediatric ACL-reconstruction**

1.	Growth plate cartilage does generally not regenerate after a drill injury.
2.	Leaving a transphyseal drill hole empty results in the formation of a bone bridge.
3.	Small bone bridges may resolve spontaneously.
4.	The formation of a bone bridge may be prevented by the transphyseal placement of a tendon graft.
5.	Permanent transphyseal hardware placement can result in a growth abnormality.
6.	A central growth plate lesion may result in a symmetric shortening whereas a peripheral growth plate lesion may result in an axial deformity.
7.	The critical size for a growth abnormality due to a central growth plate lesion is 7-9% of the size of the growth plate.
8.	The critical size for a growth abnormality due to a peripheral growth plate lesion is 3-5% of the circumference of the growth plate.
9.	The size of the growth plate injury increases with drilling obliquity.
10.	The risk of a growth deformity is inversely proportional to the remaining growth potential.
11.	The force of the growth plate is associated with body weight.
12.	An excessive graft tension may lead to a tenoepiphysiodesis.
13.	During femoral tunnel drilling, iatrogenic injury to perichondral structures should be avoided.
14.	Epiphyseal and transphyseal ACL reconstructions may induce rotational deformities at the distal femur.
15.	Graft incorporation is faster in immature specimen as compared to adults.

#### The cartilage of the growth plate does generally not regenerate after a drill injury

**Figure 3:**
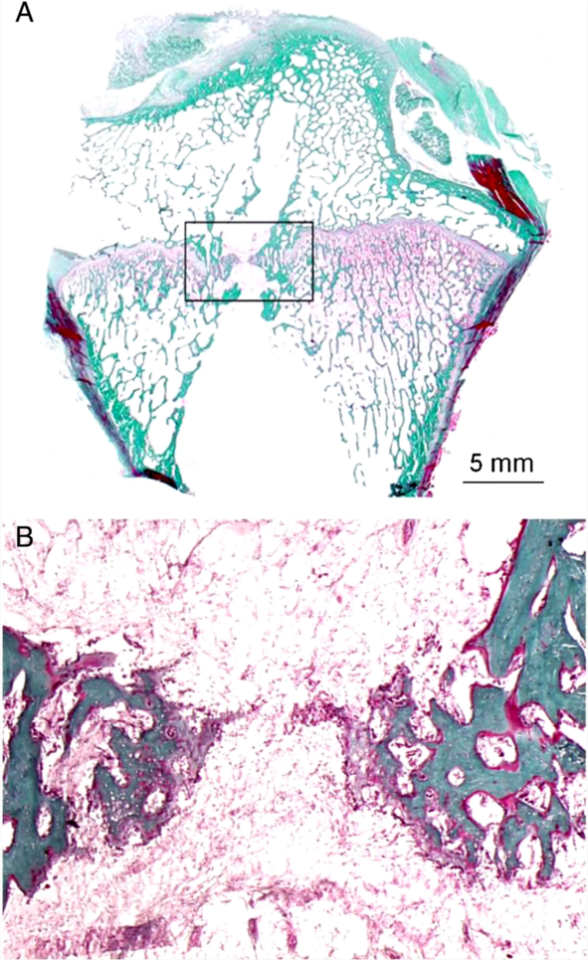
Sagittal section through a proximal tibia after drilling of a 5 mm tunnel in 4 months-old sheep. Six months later the growth plate cartilage did not recover and a bone bridge occurred. Below: Magnification X 25 (Masson-Goldner staining).

Based on their animal experiments in rabbits and pigs, Langenskiöld and colleagues have shown between the 1950’s and the 1980’s that central lesions of the growth plate had the capacity to regenerate in very young animals if a transphyseal bone bridge formation was prevented (Langenskiöld (1993); Langenskiöld 1987; Langenskiöld 1986; Langenskiöld 1981; Langenskiöld 1975; Langenskiöld 1979). In their experiments, regeneration took place by interstitial proliferation of cells from the germinal layer of the adjacent uninjured parts of the growth plate. The extent of this process was inversely proportional to the size of the used animal species. However, if a bone bridge developed after a transphyseal drill injury, the lesion of the growth plate cartilage was irreversible. This could be shown in the 1950’s by Siffert (1956) et al. in 6 weeks old rabbits. Despite this bone bridge, longitudinal growth often did not get altered. Similar findings were repeatedly noted in many other animal experiments (Figure 3).

#### Leaving a transphyseal drill hole empty results in the formation of a bone bridge (Figure 4)

**Figure 4:**
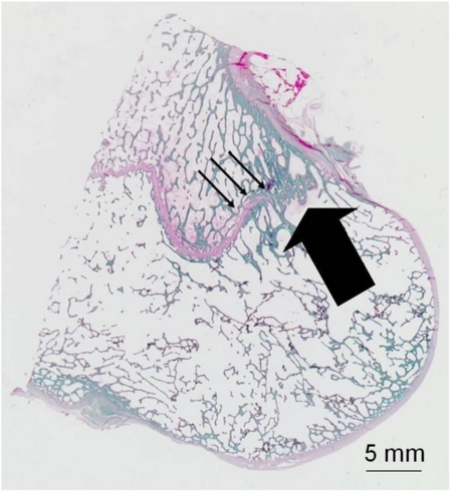
Sagittal section through the distal lateral femur of a 10 month old sheep. At 4 months a 5 mm tunnel had been drilled through the growth plate and the drill hole was not filled with a soft tissue graft. The growth plate cartilage did not recover (small arrows). A strong bone bridge occurred (large arrow) resulting in a femoral valgus deformity of 12°. Masson-Goldner staining.

Siffert (1956) analyzed the effect of a transphyseally placed 2.0 mm K-wire on the medial epiphysis of the proximal tibia in rabbits. In none of the animals growth changes could be noted. The wire grew distally from the physis and the cartilage resulting from the drill injury of the growth plate was replaced by bone. In 1960, Johnson and Southwick (1960) drilled bone tunnels (∅ 2,8 mm, corresponding to approximately 11% of the surface of the physis) through the distal femoral growth plate of 6 weeks old rabbits. In all of their 32 cases, they noticed the formation of a transphyseal bone bridge which induced a complete or a partial closure of the growth plate in 3 animals. In 21 cases the bone bridge was partially resorbed whereas resumption was complete in 8 rabbits.

These localized bone bridge formations must be differentiated from the bony transformation of the growth plate occurring at the end of the growth period. At this stage, the entire growth plate cartilage is progressively replaced by cancellous bone (Synder et al. 1994).

#### Small bone bridges may resolve spontaneously

The impact of the transphyseal bone bridge on longitudinal growth is depending on several variables. Small bone bridges may be resorbed without influencing the remaining growth process. The absence of a macroscopic growth abnormality does not mean that there are no microscopic changes of the growth plate after surgically or traumatic induced injuries. Guzzanti et al. (1994) found that microscopic changes occurred within the growth plate after injury. They found a hyperplasia of the growth plate and a loss of the classical cellular architecture of this specific cartilage. This could be confirmed by other studies (Seil 2002). Filling a bony tunnel completely by a tendon graft could not prevent the formation of a bone bridge on a systematic basis. On the contrary, an incomplete filling of the tunnel by a tendon graft of a lesser diameter did not systematically result in the formation of a bone bridge (Seil 2002).

#### The formation of a bone bridge may be prevented by the transphyseal placement of a soft-tissue graft

Stadelmaier et al. (1995) analyzed the effects of filling transphyseal bone tunnels with tendon grafts in 10 weeks old dogs. Leaving the tunnel (∅ 4 mm) empty resulted in the formation of a transphyseal bone bridge as early as two weeks postoperatively. When the tunnel was filled with a soft tissue graft bridging could be avoided. In the study the tunnels were located in the center of the proximal tibia and in the distal femur. Growth abnormalities could not be found over a four-month observation period. Janarv et al. (1998) implanted a free tendon autograft (patellar tendon or Extensor digitorum longus tendon) into distal femoral tunnels (∅ 1,7; 2,5 and 3,4 mm) in rabbits. Bone bridge developments could be diminished through the implantation of the tendon grafts and growth abnormalities could be avoided. Österman (1994) created large surgical defects in the central part of distal femoral epiphysis on 37 rabbits. If the injured physis was left without grafting, it lead to significant growth disturbance. After filling the large growth plate defect with a fatty tissue augmentation at least a partial regeneration of longitudinal growth was restored and no significant deformities of the joint contours were seen.

#### Permanent transphyseal hardware placement can result in a growth abnormality

Based on early clinical experiences with permanent or temporary epiphysiodesis (Phemister 1933; Blount and Clarke 1949) many experimental articles were published in the mid-20th century with the goal to improve growth regulation of long bones, but also to evaluate the risk of epiphyseal fractures or epiphysiolysis on the remaining longitudinal growth (Haas 1948, Haas 1945a,b, 1950; Campbell et al. 1959; Ford and Key 1956; Friedenberg 1957; Siffert 1956; Barash and Siffert 1966; Nordentoft 1969). At the end of the 20th century, first experiments were conducted with biodegradable materials. Mäkelä et al. (1987) studied the reaction of growth plates to a polyglactin 91 rod that was placed in the central portion of distal femoral growth plate in rabbits. Six weeks after implantation, the distal femoral physis showed similar growth disturbance as a drill hole which was left empty. In a later study, the same author (Mäkelä et al. 1989) showed that the size of the implant played a significant role in the development of growth disturbances. In rabbits with transphyseally placed 2.0 mm polydioxanone (PDS) implants no permanent growth disturbances could be found, whereas 3.2 mm PDS implants caused a growth disturbance similar to a drill hole. In a large animal study on sheep, placing a biodegradable interference screw over the growth plate resulted in a deformity of the proximal tibial growth plate. The fact that no major growth changes were observed may have been related to the fact that the inteference screw was placed centrally in the growth plate of the proximal tibia, similar to Stadelmaier’s study (Stadelmaier et al. 1995). This revealed that microscopic growth plate deformities may also occur with the implantation of biodegradable materials and the authors did not recommend the use of such transphyseally placed implants in pediatric ACL reconstruction (Seil 2002).

#### A central growth plate lesion may result in a symmetric shortening whereas a peripheral lesion may result in an axial deformity

In 1956, Ford and Key showed that a central lesion of the distal femoral growth plate in rabbits of a significant size could be responsible for a symmetric shortening. In one group of animals they made a simple drill hole whereas a complete destruction of the growth plate was performed in a second group of animals. The disturbance of longitudinal growth was maximal in group 2. In a third group of animals they destroyed the perichondral structures completely and found that these lesions induced severe axial deviations. In the same year Siffert (1956) drilled a 2.8 mm K-wire through the medial physis of the proximal tibia in rabbits or created an injury with a curette. He noted that that the central lesions caused decreased longitudinal growth whereas axial deformities could be seen in the group with the peripheral injuries. While these early studies documented a difference between peripheral and central physeal lesions in terms of axial malalignment, little is known about the regional differences of an injury within the physis. Since most of these experimental data resulted from small animal studies, it remains questionable whether their conclusions may be extrapolated to larger animals or even to the human knee. Nevertheless, animal experiments (Seil et al. 2008; Edwards et al. 2001) and clinical reports on complications (Koman and Sanders 1999; Robert and Casin 2010; Kocher et al. 2002) showed that the anatomical location of a peripheral growth plate lesion (medial, lateral, anterior or posterior) might have an impact on specific growth abnormalities.

#### The critical size for a growth abnormality caused by a central growth plate lesion is 7-9% of the size of the growth plate

The cut-off point from which a bone bridge cannot be spontaneously resorbed and from which the amount of injury or the cellular destruction may create growth abnormalities has not been completely evaluated yet (Pous et al. 1979; Bonnel et al. 1984; Seil 2002). A first attempt to quantify the critical size of the growth plate injury susceptible to create a growth abnormailty was undertaken by Johnson and Southwick (1960) in a rabbit model. They estimated it at 11% of the physeal surface. Nordentoft (1969) estimated the diameter of a bone tunnel to 20% of the width of the physis in the frontal plane which corresponded to approximately 10% of the physeal surface. Mäkelä et al. (1988) evaluated the size of a growth plate lesion of 2 respectively 3,2 mm bone tunnels at 13% (20%) of the width of the distal femoral physis in the frontal plane and 3% (7%) of the physeal surface. The larger diameter tunnels created growth abnormalities with significant shortening in 19 of 20 animals, whereas only minor shortening (<0,5 mm) occurred in 3 of 20 animals in the group with tunnel diameters of 2 mm. Janarv et al. (1998) confirmed these findings in another rabbit model and postulated that the relative size of a physeal drill injury necessary to cause growth disturbance starts at an injury of 7-9% of the physeal surface. Similarly, Garces et al. (1994) found no growth disturbances with surface lesions of 3,2% in the rat model.

In the human knee, Janarv et al. (1998) measured the surface of the tibial growth plate and estimated that a drill injury with a diameter of 8 mm corresponds to a lesion of 3-4% of the physeal surface. Using an MRI module for adolescent ACL reconstructive surgery (MAARS), Kercher et al. (2009) confirmed this finding in 10–15 years old children. Their module allowed for creating a 3-dimensional representation of the distal femoral and proximal tibial physis. They found that a variation of graft diameter from 6 mm to 11 mm increased the volume percent removed from 2.3% to 7.8%, corresponding to which approximately 1.1% for every 1 mm increase in tunnel diameter.

#### The critical size for a growth abnormality caused by a peripheral growth plate lesion is 3-5% of the circumference of the growth plate

Little has been published so far on this specific subject. In contrast to the amount of injured central growth plate, the minimum size of a lesion of the perichondral structures causing a localized growth disturbance or even a complete growth arrest has not been completely investigated yet. In a previous animal study on 4 months old sheep, we obtained a severe malalignment of the distal femoral physis by drilling a 5 mm tunnel through the perichondral structures and by avoiding to fill the tunnel with a tendon graft. An MRI analysis of the injured bones at 10 months revealed that the injured size of the perichondral structures was 3-5% of the size of the entire perichondral circumference (Seil 2002). Further studies need to confirm these data and to evaluate more precisely at which time point of the growth period and from which amount a localized peripheral growth plate injury may become deleterious for further longitudinal growth.

#### The size of the growth plate injury increases with drilling obliquity

**Figure 5:**
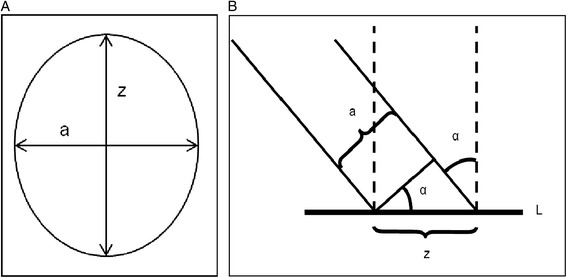
a: The inclination of a round drill leads to an elliptic drill injury (a = drill diameter; z = longitudinal diameter of drill injury proportional to the drill inclination). b: Representation of the calculation of the longest diameter of an ellipse (a = drill diameter; α = angle between tunnel and growth plate [L]; z = length of the elliptic drill injury).

If the growth plate is perforated perpendicularly during tunnel drilling, the drill injury is round and its size can be deducted from the following formula (Figure 5a and b):

$$ \begin{array}{c}A=\pi x\kern0.5em {r}^2,\\ {} where\kern0.5em r=a/2.\end{array} $$

However, the drilled tunnel is almost never perpendicular to the growth plate, leading to bigger drill injury to the growth plate than the actual diameter of the drill. Seil (2002) showed an elliptical deformation of the round drill canal with the increase of the inclination angle (Figure 5 a and b). The area of the ellipse could be calculated with following formula:

$$ \begin{array}{c}z=a/ \cos \alpha \\ {}A=\pi \kern0.5em x\kern0.5em \left(a/2\right)\kern0.5em x\kern0.5em \left(z/2\right)\\ {}=1/4\kern0.5em x\kern0.5em \pi \kern0.5em x\kern0.5em a\kern0.5em x\kern0.5em z\\ {}=1/4x\kern0.5em \pi \kern0.5em x\kern0.5em a\kern0.5em x\kern0.5em \left(a/ \cos \kern0.5em \alpha \right)\\ {}=\left(\pi \kern0.5em x\kern0.5em a2\right)/4\kern0.5em  \cos \kern0.5em \alpha \end{array} $$

In the following this theoretical example will be transposed to the femoral growth plate for either transphyseal or anteromedial portal drilling: The angle between the inclination of the drill and the femoral tunnel with transtibial drilling is approximately 25°, whereas it increases to 75° with drilling through the anteromedial portal. Considering a tunnel diameter of 7 mm, the former will result in a growth plate injury of 42 mm^2^, whereas it will result in a 3-fold increase of 148 mm^2^ with the latter. Increasing the tunnel diameter to 9 mm will result in a growth plate injury of 70 or 245 mm^2^ respectively. In terms of volume of the growth plate Kercher et al. showed in a specific MRI module that increasing the tunnel drill angle from 45 degrees to 70 degrees decreased the volume percent removed from 4.1% to 3.1%, which averages 0.2% removed for each 5 degrees increase in drill angle. These examples show that both inclination and tunnel diameter are critical.

#### The risk of a growth deformity is inversely proportional to the remaining growth potential

**Figure 6:**
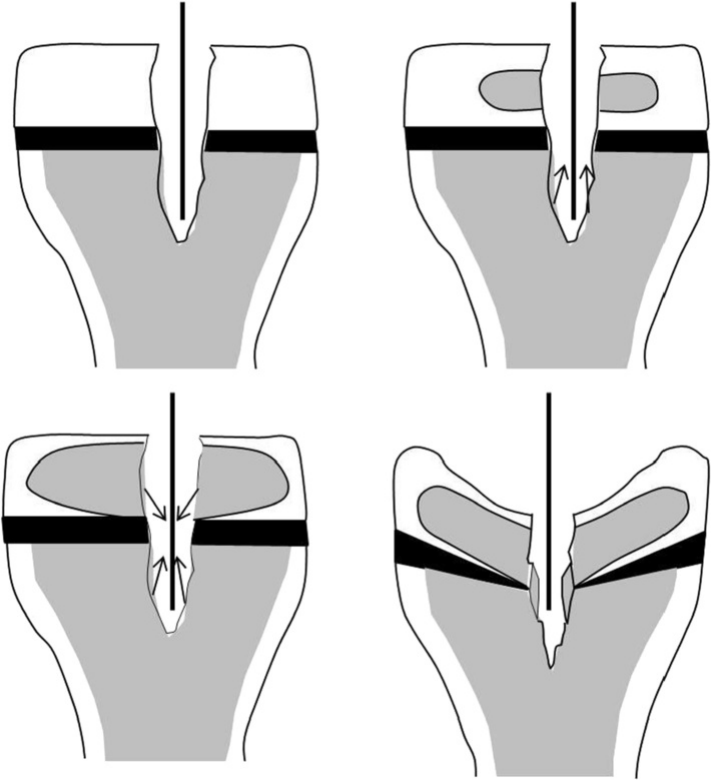
In the absence of an epiphyseal bone core or at the beginning of its formation (above), and the risk to develop a transphyseal bone bridge is lower in comparison to a situation where the bone core has developed further. In these cases a transphyseal bone formation with the development of a dip deformity could be observed (below) (modified from Barash and Siffert 1966).

The probability of a bone bridge development is supposed to be higher in older children in whom the activity of the growth plate has already decreased. It is well known that severe growth arrests may be transitional in younger children in whom the large remaining growth potential may counteract a strong localized bone bridge. In these cases, the growth plate is able to develop a sufficient force to break a bone bridge early on (Ford and Key 1956; Friedenberg 1957; Campbell et al. 1959; Nordentoft 1969; Pous et al. 1979). A similar process can be deducted from animal experiments performed by Barash and Siffert (1966)). They splitted the distal radial epiphysis cats and dogs and interposed a synthetic membrane. At the time of operation the animals were aged from several days to 8 ½ weeks. The trend to develop a bone bridge was highest in those animals with the largest epiphyseal bone core and hence the smallest distance between this epiphyseal bone core and the metaphyseal bone (Figure 6). In these animals a dip deformity of the growth plate could be documented. 5.

As a consequence of these findings one could assume that due to the low remaining growth potential in older patients with little growth remaining, the risk of a significant growth deformity is also reduced in these patients. In fact, adolescents are at a higher risk of epiphysiodesis but with little clinical consequences in terms of growth disturbances, whereas young children are at a lower risk of epiphysiodesis but sometimes with dramatic clinical consequences if the physeal bridge persists and continues to develop until the end of growth. Pediatric ACL reconstructions, and especially the transphyseal technique, in adolescents close to knee skeletal maturity may not be a benign procedure and should be performed with great care in this period.

#### The force of the growth plate is associated with the body weight

Friedenberg (1957) resected the periphery of the growth plate and its neighboring bone. He noted not only the formation of bone bridges over the resected area but also a frequent resumption of longitudinal growth. This indicated that the bone bridge may be resorbed by the distraction forces induced by the cartilage of the growth plate. An attempt to quantify this distraction force was made by Strobino et al. (1952). They found that the force which was necessary to cause a growth arrest of the bovine proximal tibial growth plate approximated 11,5 to 19,2 mg/mm^2^. In rabbits this amount seems to be much higher (15 g/mm^2^) (Bonnel et al. 1984). By analyzing the resistance of bone cement which was used for tumor prosthesis in 7–13 years old children, Safran et al. (1992) estimated the force induced by the growth plate to 58 kg per square centimeter.

#### An excessive graft tension may lead to a tenoepiphysiodesis

Ono et al. (1998) performed ACL reconstructions with iliotibial tract tendons in 25 rabbits and with patellar tendons in 31 rabbits. Despite the fact that most of the grafts were ruptured, significant growth abnormalities with limb shortening and axial deformities could be found in 10 animals in which the grafts remained intact. It was concluded that these abnormalities were secondary to the high tensioning of the grafts. The authors created the term tenoepiphysiodesis which describes the fact that an excessively tensioned graft which is thoroughly fixed both proximally and distally may be able to create compression forces on the growth plate and slow down the growth process according to the HUETER-VOLKMANN-principle (Hueter (1862); Volkmann (1862); Volkmann (1869)). This principle showed that an increasing pressure on the physis might inhibit longitudinal growth and vice versa. A similar effect could be found by Edwards et al. (2001) who performed ACL reconstructions with autologous fascia lata grafts on twelve 8 weeks old dogs. The authors tensioned their fascia lata autografts to 80 N and found a significant femoral valgus deformity. In these animals limp shortening could be found on the lateral femoral and the medial tibial side. Even if the ideal tension of ACL grafts is unknown to date, it is reasonable to believe that a tension of 80 N is excessive in skeletally immature patients. In our own findings we could show that an intraoperative tensioning of Achilles tendon autografts to 40 N did not result in major growth disturbances (Seil et al. 2008). Similar clinical complications in pediatric patients could be described by Kocher et al. (2002) and Robert and Casin (2010)). Both found a valgus deformity after ACL reconstruction in children. Similarly, placing a transphyseal synthetic graft material may lead to the same type of complication (Kurosaka 2013).

Following the description of the HUETER-VOLKMANN principle, numerous clinical examples which were reinforced by further experimental work among else by Bonnel et al. (1984), showed a rebound effect of longitudinal bone growth. This means that a transitional stimulation of longitudinal growth may occur after the removal of a supra physiological constraint. Chudik et al. (2007) found a significant increase in longitudinal growth compared to the control side in the tibia in transphyseal ACL reconstructions in dogs. This longitudinal overgrowth process can also be observed on a regular basis in children, be it in association with tibial fractures or with pediatric ACL reconstructions and may be transient (Chotel et al. 2010). So far it is not clear to which extent rebound and overgrowth effects are related to each other.

#### Iatrogenic injury to perichondral structures should be avoided

**Figure 7:**
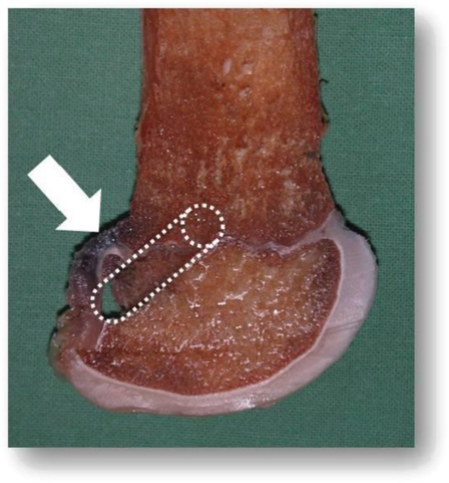
Specimen of a 10-years old girl: The arrow marks the perichondral ring of Ranvier. The dotted cylinder represents the posterolaterally located transphyseal femoral bone tunnel with a 5-mm diameter.

We analyzed the risk of growth abnormalities after injuries of the perichondral structures of the distal femur in sheep (Seil et al. 2008). In those cases in which the perichondral structures were injured and the bone tunnel was left empty, a strong valgus deformity could be observed. On the contrary, if the ACL reconstruction procedure was technically correct with extracortical graft fixations and bone tunnels which were filled with autologous tendon grafts, no growth changes could be found even if the perichondral structures were injured. A similar development could not be observed on the tibial side, indicating that the femoral tunnel has the potential to cause more severe growth abnormalities than the tibial tunnel. (Seil et al. 2008). Figure 7 illustrates the critical location of the femoral tunnel in transphyseal pediatric ACL reconstructions in a specimen of a 10-year-old girl. The transphyseal tunnel was drilled with an adult drill guide with an offset of 5 mm. This creates an eccentrically located bone tunnel, which is very close to the peripheral perichondral structures. Not filling this peripheral tunnel with a tendon graft may create a large bone bridge gapping the perichondral structures with a resulting significant valgus deformity (Seil et al. 2008).

#### Epiphyseal and transphyseal ACL reconstructions may induce rotational deformities

Chudik et al. (2007) studied the reaction of growth in rapidly growing 10-week-old dogs with three different variations of femoral drill holes. They used a central tibial tunnel and three variations of femoral graft placement: epiphyseal, over the top, or transphyseal. All techniques had a high rate of graft failure and none of them was shown to be superior. There were no significant differences in femoral longitudinal growth. The epiphyseal technique reproduced the anatomic position of the ACL graft most closely but resulted in the development of rotational deformities, a growth abnormality that had not been reported before. MRI revealed more chondral and subchondral damage on the lateral femoral condyle with the epiphyseal technique. Further investigation is needed to understand this observation and its clinical implications. So far, this deformity has not been described in the human knee.

#### Graft incorporation is faster in immature specimen

There are several experimental studies analyzing the graft incorporation in animals with open growth plates. Meller et al. (2008a,b, 2009a,b) found that in immature sheep Sharpey-like fibers develop after 3 weeks post operatively at the passing site of the graft. The maximum load to failure was decreased to 37.8 +/− 17.8 N after 3 weeks, but restored to 522.9+/− 113 N after 24 weeks. At 24 weeks, the knee had regained 69% of its biomechanical strength. The authors could demonstrate that the remodeling of the soft tissue graft is faster and more complete in immature sheep than in mature sheep. Further biological investigations were performed by Magarian et al. (2011) who studied the age related function of ACL-fibroblasts. They could prove that cellular migration and proliferation of human ACL-fibroblasts is age determined in a similar way as described in large animal models. The findings indicate that immature patients are more likely to benefit from tissue engineering than adolescent or adults. This presumption needs to be still verified in future studies. Murray et al. (2010) did their research on minipigs that concluded the better and faster healing of soft tissue in immature individuals. The research group from Murray (Murray 2009, Fleming et al. 2009) has done its work over better understanding the ACL healing in biological-cellular level. They could show promising results in minipigs after using Collagen-platelet composites around the standard ACL graft. After a dissected ACL was sutured and grafted with CPC, the control group was treated with suture alone. Load at yield, maximum load and ACL tangent modulus were all significantly higher in the suture repairs augmented with collagen-platelet composite than in repairs performed with suture alone. This work confirms the bigger biological potential of pediatric soft-tissues as compared to adults. However, its clinical relevance in the field of pediatric ACL reconstruction is still under investigation (Proffen et al. 2013).

### Summary

The risks related to different techniques of pediatric ACL reconstruction are increasingly recognized and scientific research in the field is growing. In the last decade it has been shown that a technically correct pediatric ACL reconstruction has little risk in creating growth abnormalities. Animal experiments support this hypothesis. They have helped to identify specific risk factors related to pediatric ACL reconstruction. Nevertheless, the understanding of the pathophysiologic changes of an iatrogenic injury to the growing cartilaginous structures in the knee is still incomplete and the gained knowledge in animals cannot be fully generalized in humans. Further knowledge could be gained from animal experiments by assessing complete follow-ups until the end of the growth period.
